# Editorial: Microbiomics in food security: paradigm shift in omics

**DOI:** 10.3389/fmicb.2023.1292293

**Published:** 2023-11-28

**Authors:** Anukool Vaishnav, Shekhar Jain, Devendra Kumar Choudhary

**Affiliations:** ^1^Department of Biotechnology, GLA University, Mathura, Uttar Pradesh, India; ^2^Faculty of Life Sciences, Mandsaur University, Mandsaur, India; ^3^Amity Institute of Microbial Technology, Amity University, Noida, India

**Keywords:** climate change, bio-control, soil microbes, sustainable agriculture, food security

Climate change is a major challenge for the agricultural sector in meeting the goals of food security and zero hunger. An increasing concern among agricultural scientists is protecting crop plants from the negative impacts of climate change activities, such as abiotic stresses and attacks by plant pathogens (Singh et al., 2023a). As a result, there is a pressing demand for eco-friendly technologies to safeguard crops against stressors and enhance field productivity, ultimately meeting the qualitative and quantitative needs of food consumers (Banerjee and van der Heijden, 2023). Relying solely on chemical pesticides poses long-term hazards for applicators, consumers, and pollinators, indirectly affecting the entire ecosystem. Excessive use of chemical pesticides can lead to the development of resistance in plant pathogens, including fungi and insects, making them even more destructive during disease outbreaks (Riedo et al., 2023; Wen et al., 2023). To address this challenging situation, a promising and environmentally friendly innovation is the use of soil microbes in the form of biofertilizers and biopesticides (Anand et al., 2022; Singh et al., 2023b). Additionally, the modulation of the rhizosphere microbial community is a well-documented approach for enhancing plant growth, yield, and immunity to combat various stress conditions (Singh and Vaishnav, 2021, 2022).

Over the past decade, microbe-based approaches for crop production have garnered significant attention in agricultural practices. Microbial products are safer options compared to agrochemicals (fertilizers and pesticides) for improving crop yield and nutritional value. Soil microbes play a crucial role in soil health, which is often adversely affected by the application of chemical pesticides in the field (Walder et al., 2022). In recent years, substantial research has been conducted by soil microbiologists to identify beneficial microbes for plants and understand their interactions with soil and host plants under adverse conditions (Edlinger et al., 2022; Jaiswal et al., 2022; Choudhary et al., 2023). The central theme of this Research Topic is to underscore the role of soil microorganisms in food security. This Research Topic offers a comprehensive overview of recent progress in plant-soil-microbe interactions and microbe-based formulations, such as mycorrhizal fungi and plant growth-promoting rhizobacteria, to establish a foundation for future research in this field. In this Research Topic collection, there are six research articles and three review articles covering the aforementioned topics.

Genome analysis of plant-associated bacteria is one of the most evaluated aspects, with three out of nine focusing on this field. This area is crucial for understanding the molecular mechanisms of bacterial interactions with host plants. One paper presents a whole genome analysis of *Fusarium udum*, a pathogen causing wilt in pigeon peas. *De novo* assembly identified a total of 16,179 protein-coding genes, with 1,060 genes (6.55%) identified as pathogenic genes involved in virulence. Moreover, different effector proteins related to cell wall degradation, pectin degradation, and host cell death were discovered. Comparative analysis revealed five common effector genes among all *Fusarium* species and one effector gene, *SIX* (Secreted in Xylem), that was validated in *F. udum* through wet lab experiments (Srivastava et al.). Whole genome analysis is also a promising approach for understanding the nature of stress-tolerant plant growth-promoting bacteria (PGPB) and their interactions with host plants to alleviate plant stress. *Pseudomonas aeruginosa* DJ06, for example, demonstrated PGP activities and successfully colonized sugarcane tissues. Complete genome sequencing of the DJ06 strain revealed a series of genes related to PGP properties and abiotic stress tolerance. In plant experiments, DJ06 strain inoculation promoted plant growth and biomass and regulated phytohormones in sugarcane (Guo et al.). Exploring the genomes of extremophiles helps understand their survival mechanisms under extreme conditions and their potential as sources of industrially important enzymes. In this context, a systematic genome analysis was conducted for *Virgibacillus halodenitrificans* ASH15, a halophilic bacterial isolate. The ASH15 strain exhibited survival and active PGP properties at salt concentrations of up to 25% (w/v) NaCl. Genome analysis revealed that a significant portion of genes was related to the synthesis of compatible solutes (glycine, betaine, ectoine, hydroxyectoine, and glutamate), which are known mechanisms of salt tolerance in bacteria. Interestingly, the ASH15 strain showed diverse genes related to antibiotics, CRISPRs, and medicinal compounds (including squalene) (Sharma et al.). Deciphering the whole genome of microorganisms is instrumental in understanding their interactions with their environment and serves as a source of novel compounds.

Plant stresses, both biotic and abiotic, pose significant challenges to achieving sustainable agricultural production. Plants host a diverse microbial community in their holobiont, aiding in their survival under stressful conditions (Vaishnav et al., 2014). Current efforts are focused on modulating this microbial diversity to enhance crop resilience. In this Research Topic, three papers are related to microbial-mediated alleviation of plant stress. Malviya et al. advocate for the use of mycorrhizal fungi (AMF) to control root-knot nematode infections in rice plants. The study describes the multifaceted effects of *Funneliformis mosseae, Rhizophagus fasciculatus*, and *Rhizophagus intraradices* in inducing plant defense responses against *Meloidogyne graminicola*, the root-knot disease-causing agent in *Oryza sativa*. Inoculating AMF strains also promotes nutrient uptake in rice plants, making AMF a biocontrol and plant growth-promoting agent for rice cultivation under normal and biotic stress conditions. On the contrary, the focus of another study is on antimicrobial compounds released by biocontrol microbes, which can be used directly in formulations for field applications where microbes cannot survive under adverse conditions. In this context, a study reported on the ability of *Bacillus subtilis* BS-58 as an antagonist for two devastating phytopathogens, *Fusarium oxysporum* and *Rhizoctonia solani*. Microscopy analysis revealed that BS-58 secretes antimicrobial metabolites, leading to perforation, cell wall lysis, and cytoplasmic disintegration in fungal hyphae. Further analysis through LC-MS and FT-IR characterized the antifungal metabolite as “macrolactin A” with a molecular weight of 402 Da. Gene analysis (*mln*) also confirmed the presence of this metabolite in the bacterial strain BS-58. In plant growth experiments, BS-58 inoculation was effective in reducing disease incidence against *F. oxysporum* and *R*. solani in *Amaranthus hypochondriacus* (Pandey et al.). Soil microbes can also be used as biofertilizers due to their nutrient solubilization activity, enhancing nutrient content in the soil for plant uptake. In this Research Topic, a study reports the biofortification of micronutrients through the application of different *Bacillus* spp. in wheat crops. A total of 42 isolates were recovered from the rhizosphere region, showing zinc solubilization activity on various zinc substrates, including zinc carbonate, zinc oxide, and zinc phosphate. 16S rRNA gene sequencing identified the bacterial strains as *Bacillus altitudinis, B. subtilis, B. megaterium, B. licheniformis, Brevibacillus borstelensis*, and *B. xiamenensis*. Under pot and field trials, these bacterial strains were found to enhance Zn content in wheat straw and grains. Such studies underscore the importance of soil microbes in improving crop yield and nutritional value (Yadav et al.).

This Research Topic features three review papers that focus on abiotic and biotic stress alleviation in plants with the help of soil microbes. Shakeel et al. discuss “Bakanae disease” in rice plants caused by *Fusarium fujikuroi*. The *F. fujikuroi* species complex secretes toxins such as fusarins, fusaric acid, moniliformin, and beauvericin, leading to yield losses in rice and posing risks to animal and human health. Different management strategies for Bakanae disease are discussed in the article, including biocontrol microbes, resistant plants, chemical fungicides, and physical approaches. The authors emphasize biocontrol strategies for completely eradicating this disease in rice fields (Shakeel et al.). Another review article highlights the importance of endophytic fungi as sources of antimicrobial metabolites. Endophytic fungi are rich sources of phenols, polyketides, saponins, and alkaloids, contributing to plant-released metabolites. Therefore, the exploration of plant endophytes is an emerging research area for the largescale and sustainable production of bioactive metabolites (Jha et al.). Another study emphasizes abiotic stress alleviation through the phyto-microbiome. Climate change activities significantly impact abiotic components of the atmosphere, such as soil salinity, atmospheric temperature, drought, and floods. Plant-associated microbes play a vital role in maintaining plant homeostasis during abiotic stress conditions. These microbes can improve host plant tolerance through various belowground and aboveground mechanisms that ultimately modulate plant immune responses (Singh et al.). A deeper understanding through omics approaches is necessary to explore the mechanisms of plant tolerance and develop climate-resilient crops (Diwan et al., 2022).

In summary, the results of the studies and reviews mentioned above provide a substantial amount of new and relevant data on soil and plant-associated microbes and their roles in improving plant growth, stress tolerance, and yield productivity in a sustainable manner. Despite the existing literature on this topic, the articles in this Research Topic suggest that many areas still require exploration to better harness microbial activities in sustainable agricultural practices.

## Author contributions

AV: Writing—original draft. SJ: Writing—review & editing. DC: Writing—review & editing.
